# Investigating the Protective Role of Mastery Imagery Ability in Buffering Debilitative Stress Responses

**DOI:** 10.3389/fpsyg.2019.01657

**Published:** 2019-07-24

**Authors:** Mary Louise Quinton, Jet Veldhuijzen van Zanten, Gavin P. Trotman, Jennifer Cumming, Sarah Elizabeth Williams

**Affiliations:** University of Birmingham, Birmingham, United Kingdom

**Keywords:** anxiety, confidence, sport imagery ability, coping, control

## Abstract

Mastery imagery has been shown to be associated with more positive cognitive and emotional responses to stress, but research is yet to investigate the influence of mastery imagery ability on imagery’s effectiveness in regulating responses to acute stress, such as competition. Furthermore, little research has examined imagery’s effectiveness in response to actual competition. This study examined (a) whether mastery imagery ability was associated with stress response changes to a competitive stress task, a car racing computer game, following an imagery intervention, and (b) the effects of different guided imagery content on pre-task cognitive and emotional responses. In Session 1, 78 participants (*M* age = 20.03 years, *SD* = 1.28) completed ratings of pre-task anxiety intensity and direction, confidence, and perceived control. Imagery ability was also assessed before completing the task. In Session 2, participants were randomly allocated to an imagery condition (positive mastery, negative mastery, relaxation) or control group (no imagery) before completing the task and outcome measures again. For the negative mastery group, greater positive mastery imagery ability was associated with greater perceived control and perceiving anxiety as more facilitative. Furthermore, mastery imagery ability moderated the relationship between anxiety intensity and direction. Altogether, results suggest that positive mastery imagery ability may act as a potential buffer against the effects of negative images.

## Introduction

Acute psychological stress is a common occurrence in everyday life, eliciting a range of psychological (e.g., increases in anxiety) and cardiovascular (e.g., increases in heart rate) responses (Turner, 1994; Skinner and Brewer, 2004; Moore et al., 2012). Excessive stress can be detrimental toward physical and psychological health (Schneiderman et al., 2005); therefore, individuals self-regulate stress responses by modifying the symptoms of stress (e.g., relaxing) or changing the perception of these symptoms (e.g., reappraisal; Jamieson et al., 2013). Stress can be appraised as facilitative or debilitative (Crum et al., 2013). Facilitative stress responses are characterized by better task performance, greater confidence, helpful anxiety perceptions, and/or a more favorable cardiovascular profile, whereas debilitative responses can consist of poorer performance, lower confidence, hurtful anxiety perceptions, and/or a less favorable cardiovascular profile (Williams et al., 2010; Turner et al., 2014; Trotman et al., 2018b). Consequently, it is important to establish strategies to elicit more facilitative responses to stress.

Competition is a type of stress that individuals do not always try to avoid as readily as other types of stress. Thus, when developing strategies to elicit more facilitative responses to stress, considering situational factors such as the competition context may help researchers understand the stress responses experienced (Jones, 1995). For example, 30% of the population in England engage in some type of sport at least once a week (Sport England, 2016), a proportion of which would be classified as competition. Thus, in the sport setting, competition is typically not feared by individuals and is often enjoyed and actively engaged in. Unlike the clinical literature, responses to stress in the form of a competition can be more beneficial than experiencing no response (Skinner and Brewer, 2004). Indeed, although anxiety is one of the most common and debilitating responses to stress (NHS Digital, 2018), athletes often report higher anxiety levels and feeling “psyched up” to be helpful for performance in an upcoming competition (Hanton et al., 2008) and therefore do not want to reduce these levels.

In support of not simply reducing anxiety levels, Jones (1995) proposed that strategies to elicit more facilitative competitive anxiety responses should target both the intensity experienced (i.e., severity of anxiety symptoms) and the direction (i.e., facilitative or debilitative toward performance). Importantly, anxiety direction perceptions can be a stronger predictor of performance success than anxiety intensity (Chamberlain and Hale, 2007). This research suggests that interventions that regulate anxiety responses to stress in the form of competitions should focus more on the interpretation of the anxiety rather than reducing its intensity.

More positive perceptions of anxiety symptoms are thought to be influenced by perceptions of control (i.e., greater control leads to more facilitative anxiety; Jones, 1995). Furthermore, Jones et al. (2009) posit that in a motivated performance situation like competition, higher confidence and greater perceived control are associated with positively appraising stress as a challenge, which is a state characterized by more facilitative anxiety perceptions and better performance (Williams et al., 2010; Moore et al., 2012). By contrast, a threat appraisal, resulting from lower perceived control and less confidence, is associated with more debilitative anxiety perceptions and worse performance (Williams et al., 2010; Moore et al., 2012; Turner et al., 2014). Therefore, strategies for effectively regulating anxiety perceptions to competition could be focused on raising confidence and perceived control.

Imagery is a technique that can alter the intensity and perceptions of psychophysiological stress with athletes (Cumming et al., 2007; Williams et al., 2010, 2017). Given that imagery is more effective when people can image sufficiently (Williams et al., 2013), imagery ability has been identified as a key factor for effectively regulating stress (Williams et al., 2017). Imagery ability is “an individual’s capability to form vivid, controllable images and retain them for sufficient time to effect the desired imagery rehearsal” (Morris, 1997, p. 37). Mastery imagery ability—the ease with which individuals can image mastering challenging or difficult situations—has been linked to more adaptive stress appraisals and more facilitative anxiety perceptions via greater self-confidence levels (Williams and Cumming, 2012b, 2015). Thus, those with higher mastery imagery ability, who are better at regulating their anxiety through self-confidence, may be less affected by negative imagery. Additionally, recent research has found that negative mastery imagery ability—the ability to image low feelings of confidence and a lack of control—predicted anxiety intensity and negative appraisals of stress, and both positive and negative mastery imagery ability were mediators between confidence and individuals’ dispositional stress responses (Quinton et al., 2018). Altogether, this research highlights the important role played by mastery imagery ability in regulating stress. What is still unclear, however, is whether positive mastery imagery ability is associated with stress response changes to competition. Clarifying this question would advance theoretical thinking, provide clear guidelines to those with clients participating regularly in competition (e.g., sport), and encourage developing mastery imagery ability through techniques such as layered stimulus response training (LSRT; Cumming et al., 2016) for optimal performance.

Although the impact of mastery imagery ability on responses to competition stress is not yet known, hypotheses can be developed based on research demonstrating the effect of different imagery content on responses to various types of stress. Williams et al. (2010, 2017) and Williams and Cumming (2012a) studies found that imaging low feelings of confidence and control (termed threat imagery) led to the situation being perceived as more stressful, lower confidence, and more debilitative anxiety interpretations compared to imagery of feeling confident and in control of the stress (i.e., mastery type imagery) and neutral imagery. However, other findings from these studies were mixed, as one study found that a neutral script was most helpful toward regulating stress (Williams et al., 2017), whereas others found that the mastery type script was most effective (Williams et al., 2010; Williams and Cumming, 2012a). This difference is likely due to using different tasks (i.e., public speaking, dart throwing, and a competitive experience), and using an actual stress task (i.e., public speaking, dart throwing; Williams and Cumming, 2012a; Williams et al., 2017) compared to hypothetical stress (i.e., script based on previous competitive experience; Williams et al., 2010). However, research is yet to investigate imagery’s effectiveness in altering responses to actual competition, which would be important to address to recommend particular imagery types for athletes regularly participating in competition. Therefore, it would be interesting to compare a mastery script, designed to enhance confidence and control, to a relaxation script (Cumming et al., 2007) to clarify which is most effective in regulating anxiety responses to actual competition. Clarifying this question could inform evidence-based imagery interventions and help practitioners to recommend particular types of imagery for athletes who find it difficult to cope with competition stress. As the revised applied model of deliberate imagery use (RAMDIU; Cumming and Williams, 2013) proposes that imagery content for a particular function can be influenced by the situation, it is likely that the findings of this study may be in line with Williams et al. (2010) due to a similar situation (competition), and therefore it could also be feasible that the mastery script would be more effective than a relaxation script.

### Aims and Hypotheses

The primary aim was to determine whether mastery imagery ability is associated with, and moderates, stress response changes following an imagery intervention (positive mastery, negative mastery, or relaxation script). Affect imagery ability was included as a comparison imagery ability due to emotional content that is commonly associated with a stress response, such as nervousness and excitement (Williams and Cumming, 2011). Assuming the competition elicited a stress response, it was hypothesized that higher levels of positive mastery imagery ability would (a) be associated with more favorable stress responses for the positive mastery and relaxation intervention groups, and (b) be less detrimental for the negative mastery intervention group compared to those with lower positive mastery imagery ability in the same group. It was also hypothesized that (c) mastery imagery ability would positively moderate the relationship between anxiety intensity and direction at both sessions (i.e., greater mastery imagery ability would help participants perceive increased anxiety as more facilitative).

The secondary aim was to investigate how different types of imagery can alter cognitive and emotional responses to an actual competition task (state anxiety intensity and direction, state confidence, and perceived control), rather than hypothetical or different tasks used previously (Williams et al., 2010, 2017). It was hypothesized that (d) the positive script would elicit the most facilitative stress responses for the competition task and the negative script would elicit the most debilitative responses; (e) anxiety intensity would increase from Session 1 to Session 2 for the positive and negative groups, but decrease for the relaxation group; (f) compared to Session 1, anxiety would be perceived as more facilitative for the positive group and more debilitative for the negative group; and (g) confidence would increase from Session 1 for the positive and relaxation groups but decrease for the negative group.

## Materials and Methods

### Participants

Seventy-eight male undergraduate athletes (*M* age = 20.03 years, *SD* = 1.28) participated in the study with the option of gaining course credit. Only males were recruited due to sex differences in stress responses (Bale and Epperson, 2015). The sample mainly consisted of team (*n* = 48) and individual (*n* = 25) sport athletes, with the majority coming from rugby (*n* = 16), golf (*n* = 16), and football (*n* = 14). Athletes ranged in competitive levels from elite (*n* = 10), regional (*n* = 14), club (*n* = 41), to recreational (*n* = 10). Participants were healthy with no history of epileptic seizures and cardiovascular, immune, metabolic, or kidney disease, and had no current illness or prescribed medication in the last 4 weeks at the time of the study. Participants were instructed to abstain from heavy exercise and alcohol consumption 24 h before testing and from eating and drinking caffeine 2 h before testing. Following ethical approval, participants provided informed written consent after being recruited by experimenters over an 8-week period through social media, emails, and class announcements at the university where the authors are based.

### Psychological Measures

#### Mastery and Affect Imagery Ability

Participants completed the mastery and affect subscales of the Sport Imagery Ability Questionnaire (SIAQ; Williams and Cumming, 2011). Participants imaged three items reflecting positive mastery content (staying positive after a setback, giving 100% effort when things are not going well, and remaining confident in a difficult situation) and three items reflecting affect content (positive emotions felt while doing sport, anticipation and excitement associated with sport, excitement associated with performing) before rating ease of imaging on a seven-point Likert-type scale from 1 (*very hard to image*) to 7 (*very easy to image*). The ratings were averaged to give one mastery and one affect imagery ability score. The internal reliability in this study was just below adequate (Cronbach α mastery and affect = 0.66 and 0.69, respectively). However, validity and reliability evidence has previously been found in support of SIAQ test scores (Williams and Cumming, 2011; Quinton et al., 2018).

#### Imagery Script Evaluation

Six items evaluated the generated imagery on 7-point or 10-point Likert-type scales (Cumming et al., 2007). Two items asked how easily and vividly participants could image the scripts (1 = *very hard/no image at all*, 7 = *very easy/perfectly clear*). One item asked the extent to which participants were engaged when listening to the script (1 = *none of the time*, 10 = *all of the time*). Two items assessed how imagery was perceived to impact confidence and anxiety intensities (1 = *decreased confidence/anxiety symptoms a lot*, 7 = *increased confidence/anxiety symptoms a lot*). The final item assessed how imagery was perceived to influence anxiety symptom interpretation (1 = *anxiety viewed as being much more hurtful*, 7 = *anxiety viewed as being much more helpful*).

#### State Anxiety and Self-Confidence

The Immediate Anxiety Measurement Scale (IAMS; Thomas et al., 2002) assessed cognitive and somatic anxiety intensity and direction and self-confidence in relation to the task. Participants were provided with definitions of these constructs to ensure understanding. Participants rated the extent to which they felt cognitively anxious, somatically anxious, and self-confident on a seven-point Likert-type scale from 1 (*not at all*) to 7 (*extremely*) before indicating how they perceived these symptoms from -3 (*very debilitative/negative*) to +3 (*very facilitative/positive*). Validity and reliability evidence has been found in support of IAMS test scores (Thomas et al., 2002).

#### Perceived Control

A single item assessed perceived control prior to completing the task, asking “how much control do you think you will have over the outcome of the task?” Participants responded on a seven-point Likert-type scale from 1 (*none*) to 7 (*total*).

#### Task Evaluation

Three items assessed the level of task stressfulness, difficulty, and effort experienced (e.g., Williams et al., 2017). Ratings were made on a seven-point Likert-type scale from 1 (*not at all stressful/not at all difficult/did not try at all*) to 7 (*extremely stressful/extremely difficult/tried throughout the whole task*).

### Cardiovascular Measures

Heart rate (beats per minute; bpm) was measured as a manipulation check to ensure the competition task elicited a stress response. Heart rate was recorded continuously using the Vrije Universiteit Ambulatory Monitoring System (VU-AMS5fs, TD-FPP, Amsterdam, Netherlands; De Geus et al., 1995; Willemsen et al., 1996). The VuAMS5fs used seven Ag/AgCl spot electrodes (Invisatrace, ConMed Corporation), three of which recorded electrocardiography (ECG). The ECG was recorded using three electrodes: below the right collar bone 4 cm to the right of the sternum, between the lower two ribs on the lateral right-hand side, and at the apex of the heart on the left lateral margin of the chest. Following automated R-peak detection, the interbeat interval signal was visually inspected and corrected if necessary.

### Competition Task

The competition task was the car racing computer game Need for Speed: Underground (Electronic Arts Games). The primary objective was to win a car race in the quickest time possible against three computer-controlled opponents, while avoiding traffic and other obstacles. Game manipulations allowed the computer opponents to match the ability of the participant to ensure there was never a clear win or loss. To enhance task competitiveness, a leaderboard was displayed in the lab and participants were informed that the fastest time (for each session) at the end of the study would be awarded a £10 voucher. Pre-recorded instructions informed participants about the keypad controls, that their race position would be displayed throughout the race, and that they would have one practice lap (Session 1 only) before completing the three-lap race. The experimenters provided participants with verbal encouragement throughout (e.g., Veldhuijzen van Zanten et al., 2002). The conditions for both races were pilot tested and similar in difficulty but included a different car and track than Session 1 to ensure the novelty of the task was maintained. This task has been used as a competition task in previous research and was valid for eliciting a stress response (Trotman et al., 2018b).^1^

### Imagery Scripts

The three imagery scripts (positive mastery, negative mastery, and relaxation) described the moments prior to the task, including cognitive and physiological responses. Scripts were based on those previously employed (Cumming et al., 2007; Williams et al., 2010) and included characteristics of positive and negative mastery imagery (Quinton et al., 2018). Scripts included stimulus (e.g., “you look around and notice the experimenters watching you”), response (e.g., “your heart is beating faster than usual”), and meaning (e.g., “…but you feel ready”) propositions (Lang, 1979). Scripts were pilot tested but no further changes were made. All three scripts were matched in terms of the amount of content and script length and lasted approximately 3 min. The scripts were audio recorded and played on an mp3 player.

The positive and negative mastery scripts were matched for stimulus and response propositions and described how participants would cope with the task based on theories from the stress literature (Blascovich and Mendes, 2000; Jones et al., 2009). For example, altered meaning propositions were attempted through manipulating perceptions of self-efficacy and control, which influence how stressful situations are appraised (Jones et al., 2009). The relaxation script was developed with the aim of making participants feel comfortable and calm prior to completing the task. The script included details about cognitions, body position, and physiological responses. This script predominantly included response propositions to focus on inducing a state of relaxation.^2^

### Procedure

#### Session 1

On arrival at the lab, eligibility criteria were confirmed and all procedures were explained to the participants. Participants were randomly allocated to an intervention group (1, 2, 3, or 4) from a randomly generated list devised by the experimenters: positive mastery (*n* = 18), negative mastery (*n* = 20), relaxation (*n* = 19), or control (*n* = 19). Session 1 was the same for all participants regardless of intervention condition.

Participants were connected to the cardiovascular recording equipment and comfortably seated where they remained throughout the session. A 15-min baseline period then ensued where participants watched a nature documentary to establish resting heart rate values. ECG recordings analyzed, in the 9th, 11th, 13th, and 15th min. Following baseline, participants were introduced to the task and completed the IAMS. Participants then completed the task, while heart rate was measured at 30 s and 2 min into the task. Participants completed the task evaluation form immediately after the task, had cardiovascular equipment removed, and were reminded about their second session.

#### Session 2

Session 2 for the control group was identical to Session 1. The protocol was also similar for the imagery groups except that on arrival at the lab, participants were provided with White and Hardy (1998) definition of imagery. Following baseline, but before participants listened to their allocated imagery script, they received LSRT (Cumming et al., 2016) from an experimenter trained in the technique to ensure they could image as clearly and vividly as possible. Next, participants received instructions for the task before listening to their allocated imagery script. Participants were instructed to image as clearly and vividly as possible in their preferred visual perspective. After listening to the script, participants completed the pre-task questionnaires and the task. Finally, participants completed measures of imagery ability, imagery perceptions, and task evaluation before the removal of equipment and being thanked for participation. Each visit lasted between 90 and 120 min.

### Data Reduction and Analyses

Data were analyzed using SPSS, including the process macro for moderation (version 24; Hayes, 2017). Data were first screened and cleaned in accordance with recommendations by Tabachnick and Fidell (2013), resulting in one participant (negative mastery group) excluded from the analysis as a result of univariate and multivariate outlier checks. Baseline measurements were averaged to give an overall baseline score for heart rate. Task scores were the average of the 30-s and 2-min values. Where dependent variables were correlated, to reduce the likelihood of a Type 1 error, multivariate analysis of variances (MANOVAs) were chosen over ANOVAs (Williams et al., 2010). Pillai’s Trace values were reported for all MANOVAs as this multivariate test is most robust (Olson, 1976). For MANOVAs including repeated measures, Greenhouse Geisser values were reported if Mauchly’s test of sphericity was violated. The probability value threshold for all analyses was set at 0.05 and 95% confidence intervals were reported. All significant effects were followed up with Bonferroni *post hoc* pairwise comparisons.

The Benjamini–Hochberg method was used to control for multiple comparisons in the analyses (Benjamini and Hochberg, 1995; McDonald, 2014). This method reduces the likelihood of Type 1 error while avoiding the loss of power associated with other alpha adjustments considered too conservative (e.g., Bonferroni; Shi et al., 2012). For each set of multiple analyses (e.g., correlations, MANOVAs), the *p*-values were ranked from smallest to largest and compared with Benjamini–Hochberg critical values at a false discovery rate of 0.05 (Benjamini and Hochberg, 1995; McDonald, 2014). This method has been used previously in laboratory-based stress-evoking research (Trotman et al., 2018a).

To verify that a stress response was elicited, two paired-samples *t*-tests examined differences in heart rate from baseline to the competition task at both sessions. To examine the extent to which mastery and affect imagery ability impacted the effects of the scripts, partial correlations (controlling for Session 1 scores) were conducted for each imagery group to investigate the relationships between mastery and affect imagery ability with Session 2 IAMS and perceived control scores. To investigate mastery imagery ability as a moderator between anxiety intensity and direction, analyses were separately conducted for cognitive and somatic anxiety using the process macro for SPSS (Hayes, 2017). To evaluate how well participants were able to image the scripts and the perceived effect on certain outcomes, a one-way ANOVA analyzed imagery script engagement, and two one-way MANOVAs analyzed ease and vividness of imaging the script, and the effect of the script on confidence, anxiety intensity, and anxiety perception.

To investigate if the different scripts influenced the task stress responses, two separate 2 Time (Session 1, Session 2) × 4 Group (positive mastery, negative mastery, relaxation, control) MANOVAs with repeated measures on the first factor were conducted to analyze differences in IAMS constructs (cognitive and somatic anxiety intensity and direction and confidence) and task stressfulness, difficulty, and effort. A 2 Time (Session 1, Session 2) × 4 Group (positive mastery, negative mastery, relaxation, control) repeated-measures ANOVA was also conducted to investigate if the scripts influenced perceived control prior to the task.

## Results

### Stress Response

Two paired-samples *t*-tests revealed the competition task elicited significant heart rate responses from baseline at Session 1, *t*(68) = −11.30, *p* < 0.001, and Session 2, *t*(66) = −8.05, *p* < 0.001. Significant results remained following the Benjamini–Hochberg correction. Heart rate was significantly higher during the competition task at Session 1 (*M* = 86.05, *SD* = 14.82) and Session 2 (*M* = 83.13, *SD* = 17.61) in comparison to the respective baselines (Session 1: *M* = 70.12, *SD* = 9.48; Session 2: *M* = 70.16, *SD* = 9.34). These data were further supported by self-report task stressfulness ratings reported below.

### Imagery

#### Positive Mastery Imagery Ability

##### Correlations

All correlations are shown in Table 1. There was a significant relationship between positive mastery imagery ability and confidence for the positive mastery group (*p* = 0.043). However, following the Benjamini–Hochberg correction, this correlation was no longer significant. For the negative mastery group, positive mastery imagery ability was positively correlated with cognitive (*p* = 0.005) and somatic (*p* = 0.016) anxiety direction and perceived control (*p* = 0.005). These results remained significant following the Benjamini–Hochberg correction. Better imagery ability was associated with more facilitative anxiety symptom perceptions in Session 2 for the negative mastery group. There were no significant correlations for the relaxation group.

**TABLE 1:** Mastery and affect imagery ability correlations by imagery group for Session 2 variables, controlling for Session 1 scores.

	**Positive mastery**	**Negative mastery**	**Relaxation**
**Variable**	**Mastery IA**		

Cognitive intensity	–0.488	0.177	–0.189
Cognitive direction	0.269	0.723^∗∗^	–0.400
Somatic intensity	–0.410	0.078	–0.029
Somatic direction	0.455	0.653^*^	–0.533
Confidence intensity	0.592^†^	0.398	–0.151
Perceived control	0.010	0.730^∗∗^	–0.351

	**Affect IA**		

Cognitive intensity	–0.001	0.085	–0.307
Cognitive direction	–0.246	–0.079	0.134
Somatic intensity	–0.102	0.175	–0.326
Somatic direction	–0.334	0.176	–0.117
Confidence intensity	–0.106	0.386	0.262
Perceived control	0.217	0.160	0.096

IA represents imagery ability. ^*^p < 0.05. ^∗∗^*p* < 0.01. ^†^No longer significant after Benjamini–Hochberg correction.

##### Moderation

**FIGURE 1:**
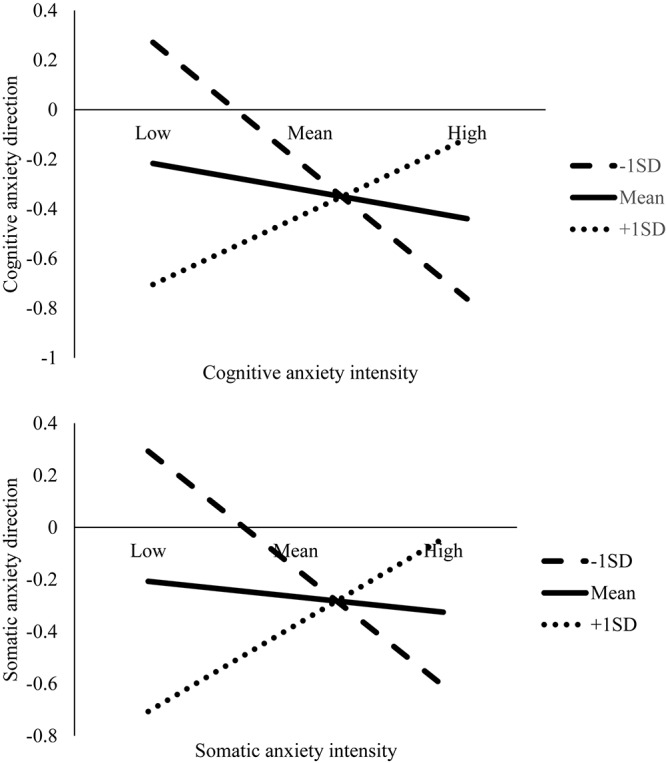
Plots for the interaction effects of cognitive and somatic anxiety intensity and mastery imagery ability on anxiety direction at Session 2.

At Session 2, mastery imagery ability moderated the relationship between cognitive [*B* = 0.24, *t*(72) = 2.31, *p* = 0.024, 95% CI (0.03, 0.45)] and somatic [*B* = 0.26, *t*(72) = 2.63, *p* = 0.01, 95% CI (0.06, 0.45)] anxiety intensity and direction. Significant results remained following the Benjamini–Hochberg correction. Graphs were then plotted to illustrate the simple slopes for low (*M* - 1 *SD*), average (*M*), and high (*M* + 1 *SD*) mastery imagery ability (Figure 1). For the low mastery imagery ability condition, there was a significant and negative relationship between cognitive [*B* = −0.30, *t*(72) = −2.22, *p* = 0.029 (−0.57, −0.03)] and somatic [*B* = −0.29, *t*(72) = −2.17, *p* = 0.033 (−0.56, −0.02)] anxiety intensity and direction. For those with lower mastery imagery ability, increased cognitive and somatic anxiety intensity was regarded as more debilitative. Although no significant relationships were found between anxiety intensity and direction for average and high mastery imagery ability (Table 2), there was a pattern for those with greater mastery imagery ability to regard increased anxiety as more facilitative (Figure 1).

**TABLE 2:** Simple slopes for differing levels of mastery imagery ability moderating between anxiety intensity and direction at Session 2.

	**Levels of mastery imagery ability**		
	**−1 SD**	**Mean**	**+1 SD**
Cognitive intensity → Cognitive direction	*B* = −0.30, *t*(72) = -2.22, *p* = 0.029 (−0.57, −0.03)	*B* = −0.06, *t*(72) = −0.66, *p* = 0.513 (−0.26, 0.13)	*B* = 0.17, *t*(72) = 1.15, *p* = 0.250 (−0.12, 0.47)
Somatic intensity → Somatic direction	*B* = −0.29, *t*(72) = −2.17, *p* = 0.033 (−0.56, −0.02)	*B* = −0.04, *t*(72) = −0.39, *p* = 0.695 (−0.23, 0.15)	*B* = 0.21, *t*(72) = 1.54, *p* = 0.128 (−0.06, 0.49)

**FIGURE 2:**
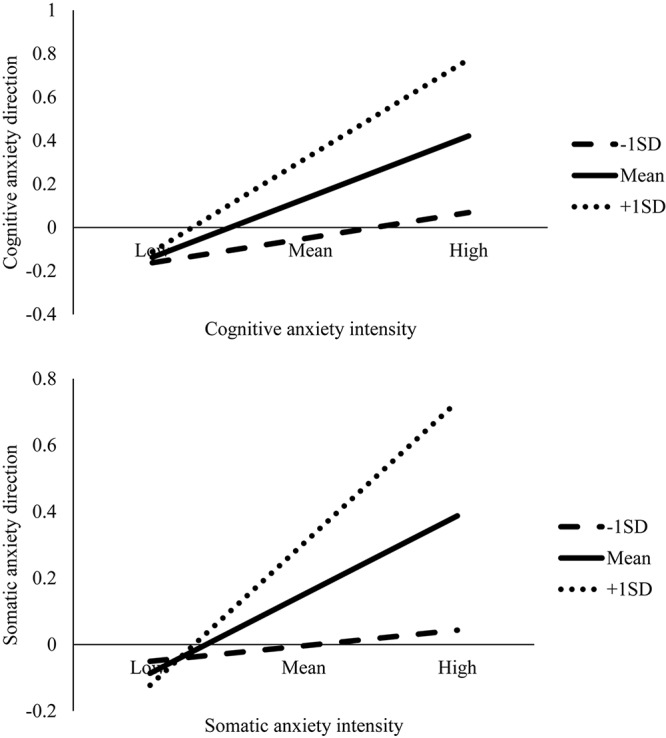
Plots for the interaction effects of cognitive and somatic anxiety intensity and mastery imagery ability on anxiety direction at Session 1.

Despite the non-significant Session 1 moderation results for cognitive [*B* = 0.12, *t*(72) = 0.84, *p* = 0.406, 95% CI (−0.17, 0.42)] and somatic [*B* = 0.15, *t*(72) = 1.18, *p* = 0.243, 95% CI (−0.10, 0.40)] anxiety, the data followed the same pattern whereby greater mastery imagery ability was associated with regarding increased anxiety as more facilitative (Figure 2).

##### Affect imagery ability

All correlations are shown in Table 1. There were no significant relationships between affect imagery ability and Session 2 variables.

##### Imagery script evaluation

Means and standard deviations are reported in Table 3. For script ease and vividness, there was a significant main effect for group at the multivariate level, Pillai’s Trace = 0.21, *F*(2, 53) = 3.09, *p* = 0.019. At the univariate level, significant group differences were for vividness, *F*(2, 53) = 5.17, *p* = 0.009, η_p_^2^ = 0.16, but not ease (*p* = 0.079). *Post hoc* analyses showed that the positive mastery group imaged their scripts significantly more vividly than the negative mastery group (*p* = 0.007). For script engagement, there was a significant difference between groups, *F*(2, 53) = 10.29, *p* < 0.001, η_p_^2^ = 0.28. The positive mastery and relaxation groups were significantly more engaged than the negative mastery group (*p* = 0.011, *p* < 0.001, respectively). For the scripts’ effect on confidence, overall anxiety, and anxiety direction for both tasks, results of the one-way MANOVA revealed that there was a significant main effect for group, Pillai’s Trace = 0.52, *F*(2, 53) = 6.15, *p* < 0.001. At the univariate level, there were significant group differences for confidence, *F*(2, 53) = 8.62, *p* = 0.001, η_p_^2^ = 0.25, anxiety intensity, *F*(2, 53) = 13.27, *p* < 0.001, η_p_^2^ = 0.33, and anxiety direction, *F*(2, 53) = 4.77, *p* = 0.012, η_p_^2^ = 0.15. The positive mastery and relaxation scripts elicited a greater effect on confidence than the negative mastery script (*p* = 0.009; *p* = 0.001, respectively). The positive and negative mastery scripts were more anxiogenic than the relaxation script (*p* = 0.008, *p* < 0.001), and the positive mastery script was perceived as more helpful for anxiety symptoms than the negative mastery script (*p* = 0.010). Significant results remained following the Benjamini–Hochberg correction.

**TABLE 3:** Means (standard deviation) for imagery evaluation items according to intervention group.

**Imagery item**	**Imagery script**		
	**Positive mastery**	**Negative mastery**	**Relaxation**
Imagery script engagement (1 = none of the time, 10 = all of the time)	7.29(1.31)^a⁣*^	5.95(1.47)	7.85(1.23)^a⁣*⁣**^
Ease of imaging script (1 = very hard, 7 = very easy)	5.29(1.11)	4.45(1.32)	5.25(0.85)
Vividness of imaging script (1 = no image at all, 7 = perfectly clear)	5.18(0.95)^a⁣**^	4.16(1.11)	4.60(0.75)
Effect on confidence (1 = decreased confidence a lot, 7 = increased confidence a lot)	5.00(0.61)^a⁣**^	4.05(1.13)	5.20(0.89)^a⁣**^
Effect on anxiety intensity (1 = decreased anxiety symptoms a lot, 7 = increased anxiety symptoms a lot)	3.76(1.15)^b⁣**^	4.37(0.90)^b⁣*⁣**^	2.70(1.03)
Effect on anxiety direction (1 = anxiety viewed as being much more hurtful, 7 = anxiety viewed as being much more helpful)	4.88(1.22)^a⁣*^	3.53(1.26)	4.20(1.44)

^*a*^Significantly greater than the negative mastery script. ^*b*^Significantly greater than the relaxation script. ^*^p < 0.05. ^∗∗^*p* < 0.01. ^∗∗∗^*p* < 0.001.

### State Anxiety and Self-Confidence

All means and standard deviations are reported in Table 4. Note that higher direction scores mean that anxiety was perceived as more facilitative. A 2 Time (Session 1, Session 2) × 4 Group (positive mastery, negative mastery, relaxation, control) MANOVA revealed a significant multivariate main effect for time, Pillai’s Trace = 0.24, *F*(5, 68) = 4.17, *p* = 0.002, and a significant time by group interaction, Pillai’s Trace = 0.42, *F*(3, 72) = 2.24, *p* = 0.006. Significant results remained following the Benjamini–Hochberg correction. Univariate analyses revealed that the main effect was for cognitive intensity, *F*(1, 72) = 12.87, *p* = 0.001, η_p_^2^ = 0.15, 95% CI (0.30, 1.05), cognitive direction, *F*(1, 72) = 9.54, *p* = 0.003, η_p_^2^ = 0.12, 95% CI (−0.85, −0.18), and somatic direction, *F*(1,72) = 10.38, *p* = 0.002, η_p_^2^ = 13, 95% CI (−0.63, −0.02). Participants had higher cognitive anxiety levels and perceived both cognitive and somatic symptoms as more debilitative at Session 2 compared to Session 1.

**TABLE 4:** Means (standard deviation) by session and intervention group.

**Imagery group**	**Session 1**	**Session 2**
	**Cognitive anxiety intensity**	
Positive mastery	2.94 (1.16)	3.72 (1.74)
Negative mastery	2.47 (1.22)	3.89 (1.82)
Relaxation	3.15 (1.57)	3.60 (1.93)
Control	3.37 (1.30)	3.42 (1.47)
Total	2.99 (1.34)	3.66(1.73)^a⁣**^
	**Cognitive anxiety direction**	
Positive mastery	0.06 (1.59)	0.11 (1.64)
Negative mastery	−0.21(1.58)	−0.74(1.41)
Relaxation	0.20 (1.51)	−0.45(1.64)
Control	0.42 (1.58)	−0.53(1.07)
Total	0.12 (1.55)	−0.41(1.46)^a⁣**^
	**Somatic anxiety intensity**	
Positive mastery	2.67 (1.28)	3.44(1.76)^a⁣*^
Negative mastery	2.42 (1.12)	3.42(1.54)^a⁣*^
Relaxation	3.15 (1.46)	2.95 (1.54)
Control	3.37 (1.17)	3.11 (1.45)
Total	2.91 (1.30)	3.22 (1.55)
	**Somatic anxiety direction**	
Positive mastery	0.67 (1.41)	0.06 (1.55)
Negative mastery	−0.21(1.51)	−0.68(1.16)
Relaxation	−0.45(1.57)	−0.30(1.46)
Control	0.58 (1.35)	−0.37(1.07)
Total	0.13 (1.52)	−0.33(1.32)^a⁣**^
	**Self-confidence**	
Positive mastery	4.17 (1.65)	4.44 (1.20)
Negative mastery	4.11 (1.20)	3.79 (1.08)
Relaxation	4.55 (.95)	4.35 (1.31)
Control	4.68 (1.38)	3.89 (.99)
Total	4.38 (1.31)	4.12 (1.17)
	**Perceived control**	
Positive mastery	5.61 (1.29)	5.50 (1.15)
Negative mastery	5.26 (1.15)	4.79 (1.40)
Relaxation	5.45 (1.00)	5.80 (1.11)
Control	5.39 (1.04)	5.50 (1.04)
Total	5.43 (1.11)	5.40 (1.22)
	**Task stressfulness**	
Positive mastery	3.44 (1.46)	3.44 (1.58)
Negative mastery	3.53 (1.02)	4.32 (1.11)
Relaxation	3.70 (1.26)	4.10 (1.25)
Control	3.17 (1.51)	3.78 (1.31)
Total	3.47 (1.31)	3.92(1.33)^a⁣**^
	**Task difficulty**	
Positive mastery	3.72 (1.36)	3.78 (1.59)
Negative mastery	4.32 (1.16)	4.32 (1.06)
Relaxation	4.05 (1.00)	4.25 (1.48)
Control	3.56 (1.42)	3.89 (1.13)
Total	3.92 (1.25)	4.07 (1.33)
	**Task effort**	
Positive mastery	5.61 (1.50)	5.67 (1.28)
Negative mastery	5.89 (1.10)	5.68 (1.25)
Relaxation	6.40 (1.05)	5.80 (1.80)
Control	6.28 (.96)	5.67 (1.28)
Total	6.05 (1.18)	5.71(1.40)^a⁣*^

^*a*^Significantly different than Session 1. ^*^p < 0.05. ^∗∗^*p* < 0.01. ^∗∗∗^*p* < 0.001.

For the time by group interaction, univariate analyses revealed that this effect was for somatic intensity, *F*(3, 72) = 3.45, *p* = 0.021, η_p_^2^ = 0.13, and approached significance for somatic direction, *F*(3, 72) = 2.55, *p* = 0.063, η_p_^2^ = 0.10. Participants in the positive mastery, *p* = 0.035, 95% CI (0.06, 1.50), and negative mastery, *p* = 0.006, 95% CI (0.30, 1.70), groups had higher somatic intensity levels at Session 2 than at Session 1. For somatic direction, there was a trend for the positive mastery and control groups to perceive their symptoms as more debilitative at Session 2 compared to Session 1. At the multivariate level, there was no main effect for group and no time by group interaction for confidence intensity, cognitive intensity, or cognitive direction.

### Perceived Control

All means and standard deviations are reported in Table 4. A 2 Time (Session 1, Session 2) × 4 Group (positive mastery, negative mastery, relaxation, control) ANOVA revealed no main effects for time, *F*(1, 71) = 0.05, *p* = 0.823, or group, *F*(3, 71) = 1.41, *p* = 0.246, and no time by group interaction, *F*(3, 71) = 1.67, *p* = 0.182.

### Task Evaluation

All means and standard deviations are reported in Table 4. A 2 Time (Session 1, Session 2) × 4 Group (positive mastery, negative mastery, relaxation, control) MANOVA revealed a significant multivariate main effect for time, Pillai’s Trace = 0.18, *F*(3, 69) = 4.63, *p* = 0.004. Significant results remained following the Benjamini–Hochberg correction. Univariate analyses revealed that this effect was for task stressfulness, *F*(1, 71) = 7.57, *p* = 0.008, η_p_^2^ = 0.10, 95% CI (0.12, 0.78), and task effort, *F*(1, 71) = 4.80, *p* = 0.032, η_p_^2^ = 0.06, 95% CI (−0.65, −0.03), but not for difficulty. Participants found Session 2 significantly more stressful, but put in significantly less effort compared to Session 1. There was no significant multivariate main effect for group, or time by group interaction.

## Discussion

The present study examined whether positive mastery imagery ability was associated with stress response changes to a competition task following an imagery intervention, while also investigating how positive mastery, negative mastery, and relaxation imagery influenced the cognitive and emotional (anxiety, confidence, and perceived control) pre-task responses. The task elicited a stress response in accordance with previous literature (Veldhuijzen van Zanten et al., 2002). Also, when considering manipulation checks, the mean values support that participants appeared motivated and engaged in the task.

A key strength of the present study, in comparison to previous research (e.g., Williams et al., 2010, 2017), is the theoretical underpinning of the RAMDIU (Cumming and Williams, 2013). The use of this framework allowed for the discovery of a new buffering role for mastery imagery ability against the debilitative effects of imagery and therefore a novel theoretical contribution to existing literature. Another strength of this study was the use of actual competition as a stress task. Competition is a unique type of stress that people approach rather than avoid compared to most types of stress studied, which means these results can contribute to the broader implications of what can be learned from a type of stress that people choose to engage in, and the strategies used to regulate such stress (e.g., mastery imagery ability).

### Key Findings and Implications: Primary Aim

In support of our hypotheses, results suggest that the imagery’s effectiveness was determined by imagery ability. In particular, for the negative mastery group, greater positive mastery imagery ability was associated with greater perceived control and a lower reduction in anxiety direction (i.e., less likely to perceive anxiety symptoms as debilitative). In other words, those in the negative imagery group with poorer positive imagery ability were more greatly impacted by their assigned imagery condition, suggesting that positive mastery imagery ability acts as a buffer against imagery eliciting debilitative stress responses (e.g., debilitative anxiety). This finding supports the RAMDIU as imagery ability influenced outcomes experienced from a stress task (Cumming and Williams, 2013). However, the novelty of our finding provides an additional theoretical contribution to this model by suggesting that imagery ability can also buffer against the debilitative effects of negative imagery, therefore extending beyond what the revised model hypothesized.

Support that mastery imagery ability acts as a buffer against negative imagery was demonstrated using moderation analyses: those with lower mastery imagery ability perceived increased levels of anxiety as more debilitative. Although the moderation relationships were not significant at Session 1, this could be explained by increased task stressfulness ratings at Session 2. At the first visit, participants were likely still acclimatizing to the laboratory conditions and learning how to perform the task. Although there were some differences introduced in Session 2 to maintain a degree of task novelty (e.g., different race track), the learning from Session 1 would enable participants to focus more on performing and the results, hence the increased ratings of stressfulness but reduced effort. That this moderation effect was significant for all participants, regardless of their condition, indicates that the stress-inducing factors of competition were strong enough to elicit an anxiety response for all groups. Moreover, this anxiety response was of a sufficient level for participants’ mastery imagery ability to exert a moderating effect. Recent research has found positive mastery imagery ability to be associated with either anxiety intensity or anxiety direction (Quinton et al., 2018; Williams et al., under review). However, the current study extends these findings by suggesting that the role of mastery imagery ability as a correlate of anxiety may be more complex than previously thought, playing a moderating role in perceiving anxiety as more facilitative. This novel finding should be explored in future research to determine its replicability and generalizability to other settings (e.g., other competitive and stress-evoking situations). If replicated, developing mastery imagery ability could be a significant strategy for promoting more facilitative anxiety interpretations during stress.

During stressful scenarios, spontaneous negative images can be experienced (Van de Braam and Moran, 2011). The present results allude to the importance of mastery imagery ability in protecting against the debilitative effects of negative images. The importance was further emphasized by the lack of any significant results with affect imagery ability. Although research shows that the ability to image intervention content can influence imagery’s effectiveness (McKenzie and Howe, 1997), this study highlights the importance of more general imagery ability, positive mastery, by demonstrating that the ability to image this content may play a role in the effectiveness of a particular imagery intervention. More broadly, findings demonstrate the importance of imagery ability impacting upon the effectiveness of imagery use and, in line with Jones (1995) framework, suggest that individual factors such as imagery ability should be considered when investigating responses to stress and how they are perceived.

Another type of imagery ability in this study, although employed as a manipulation check, could be imagery script engagement. Supported by the computational theory of imagery (Kosslyn et al., 2006), the ability to remain engaged in a script could reflect the maintenance stage of image generation. The negative mastery group was less engaged in their script, which, although could be noted as a limitation, could also imply that lower script engagement acts as a protective factor against debilitative imagery. It is possible that higher engagement with facilitative imagery could elicit more positive responses. Although engagement is crucial for imagery effectiveness in clinical settings (Steenbergen et al., 2009), scarce research has explored engagement within other settings, such as sport and competition. As debilitative imagery can be more powerful in eliciting stress responses than facilitative imagery (Nordin and Cumming, 2005), it is important to understand this relationship and what strategies (e.g., imagery rescripting) may be most effective to prevent debilitative stress responses and poor performance.

### Key Findings and Implications: Secondary Aim

In accordance with our hypotheses and previous research (Williams et al., 2010, 2017), the scripts containing positive and negative mastery content reported higher cognitive and somatic anxiety levels. However, in contrast to our hypothesis, there was a trend for anxiety to be perceived as more debilitative for the positive mastery and control groups but not the negative mastery group. These results were unexpected and also in contrast to research where participants who imaged neutral or coping-based content perceived anxiety symptoms as facilitative (Cumming et al., 2007; Williams et al., 2010, 2017) and those who imaged negative content perceived anxiety as debilitative (Cumming et al., 2007; Williams et al., 2010). Although some of these studies included hypothetical competitions or low stress-evoking situations, the scripts provided stimulus propositions based on personal experiences, which likely contributed to an increased meaning, and therefore effectiveness, of the imagery (Lang, 1979). In this study, the unexpected results could be due to the imagery of the task being less familiar compared to previous studies, and subsequently less meaningful and effective for participants. This notion is supported by the RAMDIU (Cumming and Williams, 2013), which posits that the meaning of an image influences what function (e.g., anxiety producing) the image content (e.g., positive mastery) serves. Importantly, when using positive mastery imagery, results suggest that practitioners should ensure imagery is meaningful and that it has the intended facilitative effect for actual performance scenarios.

Interestingly, additional results were also in contrast to our hypotheses and previous research. In contrast to Williams et al. (2010, 2017), Williams and Cumming (2012a) studies, there were no significant group differences for confidence or perceived control in relation to the competition task. Furthermore, although Williams et al. (2017) found that the neutral script was occasionally more facilitative than the challenge script, this was not the case for the relaxation script used in this study. These results could be due to the variation between these imagery groups in the vividness and engagement of the scripts. Although there were no group differences in ease of imaging (i.e., one indicator of imagery ability), the positive mastery group imaged their scripts significantly more vividly than the negative mastery group, and the positive and relaxation groups were significantly more engaged in their scripts than the negative group. These findings suggest that participants found it easier to image the positive script content compared to negative, which could have influenced the effect of the imagery on task responses (i.e., confidence and perceived control). Therefore, researchers and practitioners conducting imagery interventions should ensure adherence to scripts and verify during the intervention (i.e., rather than after) whether participants can sufficiently image all aspects of the scripts, providing extra training where necessary (e.g., LSRT; Cumming et al., 2016).

Findings expand on Williams et al. (2010, 2017), Williams and Cumming (2012a) research by investigating imagery’s effect on responses to actual competition, and highlights the importance of considering the situation associated with the imagery (i.e., public speaking or competition, hypothetical or real). This study supports that responses to an actual competition task are different to a real task in the form of dart throwing (Williams and Cumming, 2012a), a speech preparation task (Williams et al., 2017), and hypothetical competition (Williams et al., 2010). The collective results from these studies may demonstrate that imagery scripts (challenge or positive mastery, threat or negative mastery, or relaxation) might not be as effective for a stressful task where stimuli are constantly presented (i.e., car racing competition) and performance was evaluated, in comparison to a hypothetical task or a task which involves greater internal concentration (i.e., public speaking preparation task or dart throwing). Thus, in accordance with the RAMDIU (Cumming and Williams, 2013), the content (e.g., imagery script), situation (e.g., stress task, hypothetical or real), and individual components (e.g., positive mastery imagery ability) appear crucial to consider when implementing imagery interventions for stressful situations.

### Limitations and Future Research

Although the current study provides some important contributions to the literature, it is not without limitations. Numerous tests were run in a small sample; however, multiple comparisons were controlled for using a conservative method that allowed statistical power to be maintained (Benjamini and Hochberg, 1995). Task novelty may have been influenced by previous task experiences; thus, research should test this consideration as a confounding variable (e.g., Williams and Cumming, 2012a). Also, the competition task differed in stressfulness across sessions. Although these tasks could have been counterbalanced (e.g., race track) to rule out the order being a confounding variable, the nature of the imagery intervention meant that participants had to be exposed to the task twice and therefore it was likely that the novelty, and stress response, would be reduced. Stress research makes the issue of novelty difficult to control, as the unique aspect of stress is that it is often associated with fear of the unknown. Therefore, undertaking a task twice is likely to yield differences in the stress response. However, this difference could also be viewed as a strength as completing a task twice often results in a loss of stressfulness of the task, but in this case, the task was more stressful the second time. Future research should expand on combining imagery interventions in repeated exposures to stress tasks and the subsequent influence on the stress response experienced. Future research should also ensure daytime is controlled for between laboratory visits.

## Conclusion

Findings demonstrated that positive mastery imagery ability can determine the effectiveness of imagery’s use. Results found a new buffering role for mastery imagery ability against the debilitative effects of negative imagery (e.g., debilitative anxiety), providing a novel theoretical contribution to the RAMDIU (Cumming and Williams, 2013) and a new understanding of how this type of imagery interacts with anxiety intensity and direction. Results also suggested, in contrast to Williams et al. (2010, 2017), that the imagery type used may not be more/less beneficial for a novel computer car racing task, which may be due to the different nature of hypothetical vs. real competition experiences or competition vs. other stress tasks (e.g., public speaking). Altogether, in accordance with and extending the RAMDIU (Cumming and Williams, 2013), positive mastery imagery ability varied across individuals and acted as a buffer, which together with the situation (e.g., competition task) likely influenced what function (e.g., anxiogenic) the image content (e.g., positive mastery) served, and therefore the outcomes experienced (e.g., more debilitative anxiety interpretations). Positive mastery imagery ability should be developed to reduce the impact of debilitative imagery and maladaptive responses to stress.

## Data Availability

The datasets generated for this study are available on request to the corresponding author.

## Ethics Statement

This study was carried out in accordance with the recommendations of the University of Birmingham’s ethical review board, with written informed consent from all subjects. All subjects gave written informed consent in accordance with the Declaration of Helsinki. The protocol was approved by the University of Birmingham’s ethical review board.

## Author Contributions

MQ made substantial contributions to the study design, data collection, entry, and analysis, and drafting of the manuscript. GT made substantial contributions to the study design, data collection and entry, and provided feedback on the manuscript. JC provided critical analysis of, and feedback on the manuscript. SW and JVvZ made substantial contributions to the study design, analysis, and provided feedback on drafts of the manuscript.

## Conflict of Interest Statement

The authors declare that the research was conducted in the absence of any commercial or financial relationships that could be construed as a potential conflict of interest.

## Footnotes

1 Please note that this study marginally overlaps with the current study in terms of data (Session 1 only). However, the manuscripts are sufficiently distinct to not warrant concern.

2 Scripts can be found in Supplementary File 1.
