# Supplementary material for: Investigating the Protective Role of Mastery Imagery Ability in Buffering Debilitative Stress Responses
Source: Front Psychol. 2019 Jul 24;10:1657. doi: 10.3389/fpsyg.2019.01657 (PMC6668598; doi:10.3389/fpsyg.2019.01657)
Supplement: Supplementary file 1 [file Table_1.DOCX]

Supplement File 1 – Imagery scripts

*Positive mastery script*

You are now just a couple of minutes away from the start of the competition race… your heart is beating faster than usual and you are breathing more deeply………… the butterflies in your stomach make you realise the importance of this race……… but you feel ready…….…….the positive adrenalin and feelings of confidence you have tell you that you will perform well……… You know that to race quickly you will need to avoid hitting traffic and obstacles during the race…as these would slow you down…however, you have complete control of the car ….……and the equipment is set up to allow you to play the game without complications……you feel in control of this race……you have played the game before…… and because of this, you can feel your confidence in your own ability to perform well in the race.…………you believe that you can win the race you are about to compete in……… your heart is pumping rapidly and you can feel the blood flowing through your body…… ……although this time you will race on a different track, in a different car, you believe that you have the ability to overcome the challenge……you are using the same controls as before… you see them in front of you and you believe that you are capable of operating them effectively to control the car….which will allow you to race as fast as possible………you set yourself the aim of trying to do your best……you think of the task as a challenge…and you know you are someone capable of meeting that challenge…………you know the other cars in the game might have the ability to go faster than you……but you can feel your confidence in your own ability to perform well and race fast…… and you relish the opportunity to compete against them…………you feel the adrenalin rush through your body, reaching all of your muscles…… ……you have never experienced so many intense positive feelings prior to playing a computer game competition ………………… You look around and notice the experimenters watching you about to play the game… and savour the prospect of demonstrating your competence in front of them…………there is real potential for you to win the race in your fastest possible time….and finish with your name at the top of the leader board.

*Negative mastery script*

You are now just a couple of minutes away from the start of the competition race… your heart is beating faster than usual and you are breathing more deeply………… the butterflies in your stomach make you realise the importance of this race……… but you do not feel as ready as you would like to be……. you feel your nerves increase as you worry that you will not be able to win……… You know that to race quickly, you will need to avoid hitting traffic and obstacles during the race…as these would slow you down…but this is not under your control….…… you feel you have no control over this race……you have only played the game a few times before in these conditions, and because of this, you obviously can’t be sure that you will perform well in the race.…………you are not sure that you can win the race you are about to compete in……… your heart is now pumping rapidly and you can feel the blood flowing through your body…… ……this time you will race on a different track, in a different car, which you are not familiar with…… you believe that you are not able to overcome the challenge…… you see the control pad in front of you…but you now feel worried that you may not be able to control the car effectively…….which will stop you from racing as fast as possible……… you set yourself the aim of not performing poorly……you think of the task as a challenge… and you know you are someone not capable of meeting that challenge…………you know the other cars in the game have the ability to go faster than you……you fret about competing against them……and you cast even more doubt in your own ability to perform well and race fast…………you feel the adrenalin rush through your body, reaching all your muscles…… ……you have never experienced so many intense negative feelings prior to playing a computer game competition ………………… You look around and notice the experimenters watching you about to play the game… and you are concerned about revealing your weaknesses in front of them…………there is real potential to lose this race….and finish with the slowest time in the study.

*Relaxation script*

You are now just a couple of minutes away from the start of the competition race…………you sit quietly and gather your thoughts before you begin the race…… you feel well prepared… and are relaxed about the upcoming race………… as you rest before the competition, you feel your heart rate begin to slow down ………… you concentrate on your breathing…… and gradually reduce its rate by breathing slowly in…and then out again…………you let all other thoughts go …and focus on your breathing ……. … with each breath you take, you notice yourself becoming more and more relaxed…….feel your body gradually sinking into a state of relaxation……you remain composed…… in a state of calmness…………your body feels comfortable in the position you are sitting in……..and you feel your body continue to relax as all remaining tension gently leaves your muscles ………… your body feels at ease as you continue to become more relaxed……..your heart rate continues to fall…getting slower…and slower…………let go of any other thoughts…and remain in your own world………… your heart rate has gently dropped and your breathing rate has gradually slowed down………… any anxiety you previously experienced has completely evaporated from your body… leaving you in a state of relaxation and contentment............you feel peaceful……. your body is now at a comfortable temperature…………… your muscles are loose…….your hand that will operate the controls is relaxed………and your finger is supple………you are ready to perform the computer task………… you concentrate on your breathing for one final time………you take your time…slowly taking a deep breath in…….then as you gradually breathe out… you are aware of the complete state of relaxation your body has entered……..you feel relaxed, stress-free, and ready to embrace the competition race…
